# Insulin Receptor Isoforms A and B as well as Insulin Receptor Substrates-1 and -2 Are Differentially Expressed in Prostate Cancer

**DOI:** 10.1371/journal.pone.0050953

**Published:** 2012-12-10

**Authors:** Martin Heni, Jörg Hennenlotter, Marcus Scharpf, Stefan Z. Lutz, Christian Schwentner, Tilman Todenhöfer, David Schilling, Ursula Kühs, Valentina Gerber, Fausto Machicao, Harald Staiger, Hans-Ulrich Häring, Arnulf Stenzl

**Affiliations:** 1 Department of Internal Medicine, Division of Endocrinology, Diabetology, Angiology, Nephrology and Clinical Chemistry, Eberhard Karls University Tübingen, Tübingen, Germany; 2 Department of Urology, Eberhard Karls University Tübingen, Tübingen, Germany; 3 Institute for Pathology, Eberhard Karls University Tübingen, Tübingen, Germany; 4 Institute for Diabetes Research and Metabolic Diseases of the Helmholtz Center Munich at the University of Tübingen, Partner in the German Center for Diabetes Research (DZD), Tübingen, Germany; Universita Magna-Graecia di Catanzaro, Italy

## Abstract

**Aims/Hypothesis:**

In different cancers types, insulin receptor isoform composition or insulin receptor substrate (IRS) isoforms are different to healthy tissue. This may be a molecular link to increased cancer risk in diabetes and obesity. Since this is yet unclear for prostate cancer, we investigated IR isoform composition and IRS balance in prostate cancer compared to benign and tumor adjacent benign prostate tissue and brought this into relation to cell proliferation.

**Methods:**

We studied 23 benign prostate samples from radical cystectomy or benign prostatic hyperplasia surgery, 30 samples from benign tissue directly adjacent to prostate cancer foci and 35 cancer samples from different patients. RNA expression levels for insulin receptor isoforms A and B, IRS-1, IRS-2, and IGF-1 receptor were assessed by quantitative real-time RT-PCR. In addition, RNA- and protein expression of the cell cycle regulator p27^Kip1^ was quantified by real-time RT-PCR and immunohistochemistry.

**Results:**

Insulin receptor isoform A to B ratio was significantly higher in cancer as well as in tumor adjacent benign prostate tissue compared to purely benign prostates (p<0.05). IRS-1 to IRS-2 ratios were lower in malignant than in benign prostatic tissue (p<0.05). These altered ratios both in cancer and adjacent tissue were significantly associated with reduced p27^Kip1^ content (p<0.02). Interestingly, IGF-1 receptor levels were significantly lower in patients with type 2 diabetes (p = 0.0019).

**Conclusions/Interpretation:**

We found significant differences in the insulin signaling cascade between benign prostate tissue and prostate cancer. Histological benign tissue adjacent to cancer showed expression patterns similar to the malignancies. Our findings suggest a role of the insulin signaling pathway in prostate cancer and surrounding tissue and can hence be relevant for both novel diagnostic and therapeutic approaches in this malignancy.

## Introduction

Type 2 diabetes (T2DM) is associated with increased risk for several cancer types [1]–[3]. The risk for the most prevalent cancer in men, prostate cancer, seems to be unaltered by diabetes [4] but this is thought to be due to lower testosterone levels in T2DM [3], [5], [6]. Furthermore, survival time after diagnosis of prostate cancer is shorter in men with T2DM compared to men without [7].

For many types of cancer, alterations in the insulin signaling cascade have been reported. This starts at the level of the insulin receptor (IR). This receptor occurs in two isoforms, isoform A that is predominantly expressed prenatally and isoform B that is expressed in adult differentiated tissue [8]. While IR isoform B has a high affinity for insulin and is responsible for most of this hormone's metabolic effects, isoform A has additionally a high affinity for insulin-like growth factor (IGF)-II and contributes to cell proliferation. IR isoform A is aberrantly expressed in many cancer cells [8]. While overexpression of IR in prostate cancer and higher activity of the signaling chain downstream has been reported [9]–[11], isoform configuration in this cancer has not been studied yet.

The docking proteins downstream of IR and other receptor tyrosine kinases (e.g. IGF-1 receptor), insulin receptor substrate (IRS)-1 and IRS-2 are crucial to further communicate signals from these receptors [12], [13]. Despite IRS-1's and IRS-2's high homology, they have non-redundant functions in metabolic control and cell proliferation [14]. These docking proteins have been well characterized with regard to metabolism and diabetes but only few studies investigated their roles in malignancies [15]. The role of these proteins in prostate cancer is still unclear.

Recent studies describe molecular alterations in histologically benign tissue of prostate cancer bearing glands [9], [16]. Besides scientific interest in tumor biology, knowledge about such alterations is of great importance for clinical use, e.g. when establishing diagnosis in biopsy material or to determine surgical margins.

Aims of this study were to investigate *(i)* IR isoform composition and IGF-1 receptor expression and *(ii)* IRS balance in prostate cancer compared to benign tissue as well as to tumor adjacent benign tissue; and *(iii)* to bring these results in relation to p27^Kip1^, a well described cell cycle inhibitor in prostate cancer [17].

## Methods

We studied samples from 65 patients subjected to radical prostatectomy for prostate cancer (age 50–76 [median 65] years). Prostatic tissue from patients who underwent radical cystoprostatectomy due to bladder cancer (n = 13, median age 67 years) or transvesical/transurethral prostate-adenoma-enucleation due to hyperplasia (n = 10, median age 70 years) with no sign for prostate cancer were included. Clinical characteristics are given in Table 1. For all patients with prostate cancer, pre-operative PSA levels as well as TNM-staging and Gleason score were available. For the analysis of Gleason score, we compared samples from the ‘prostate cancer’ group with a Gleason score up to 3+4 ( = 7a; N = 11) with such having a Gleason score of 4+3 = 7b and higher (N = 24).

**Table 1:** Clinical characteristics of the patients.

Patients from whom samples are derived with…	Benign prostate	Benign tissue adjacent to prostate cancer	Prostate cancer	p
N	23	30	35	-
Age (y)	67.0±8.4	65.7±5.8	63.0±6.8	0.1
BMI (kg/m^2^)	26.3±4.7	26.7±3.6	27.1±3.7	0.6
Known type 2 diabetes mellitus (no/yes)	19 (20)/3*	26/4	30/5	0.5
Insulin or sulfonylurea therapy (no/yes)	19/4	28/2	32/3	0.4
Number of patients taking insulin/sulfonylurea/metformin/other oral antidiabetic drugs#	2/2/2/0	2/0/2/2	3/0/2/0	-

Data are given as means ±SD. BMI – body mass index;

* = 1 patient had known type 1 diabetes mellitus.

# = patients taking combination therapy are counted more than once.

The study was approved by the local ethics committee (University of Tübingen) and all participating patients gave written informed consent.

To ensure optimal quality of the prostate tissue and to avoid delayed freezing of the fresh tissue, a procedure was performed to ensure immediate freezing. Tissue from normal prostates were frozen in liquid nitrogen immediately after surgical specimen resection (‘benign’ group; N = 23). Samples were enrolled in the study, if a) histopathological work up of the whole surgical specimen revealed no sign of prostate cancer and b) a representative slide from the sampled tissue showed non-malignant prostate histology.

Tissues from radical prostatectomy specimen (resulting in the ‘tumor adjacent prostate tissues’ and the ‘prostate cancer tissues’) were sampled as follows: immediately after resection the specimen was digitally palpated and cut in at the localization of the supposed tumor area (the area of peripheral hardness). Then a peripheral sample in size of approximately 5×5×3 mm was cut out and divided longitudinally into 3 lamellas, from which the outer two (I and III) were snap frozen in liquid nitrogen. The middle lamella (II) was formalin fixed, paraffin embedded and processed to a histological slide. Workup of the middle slide by an experienced uropathologist decided the group affiliation of the parallel frozen tissue: if there was no sign of prostate cancer on the slide, the respective tissue was led to the ‘tumor adjacent benign prostate tissue’-group (N = 30). If the slide revealed tumor tissue below 60% of the area, the respective tissue was excluded from the study because of its mixed tissue characters. If the tumor proportion was above 60% of the slide, the parallel tissue was included into the analysis as ‘prostate cancer tissue’ (N = 35).

Due to this approach, each analyzed sample was derived from a different patient.

The frozen tissue samples were used for RNA isolation, the corresponding paraffin tissues for immunohistochemistry. The frozen samples were lysed in RLT using Tissue Lyser kit (Qiagen). RNA was extracted by RNeasy Mini Kit (Qiagen). PCRs (in duplicates) were performed on a LightCycler 480 (Roche Diagnostics) using Probes Master and fluorescent probes from the Universal Probe Library (Roche Diagnostics). The following real-time PCR protocol was used [18]:

Denaturation program (95°C for 5 minutes), an amplification and quantification program repeated 45 times (95°C for 10 seconds, 60°C for 30 seconds, 72°C for 1 second [fluorescence acquisition]), and finally a cooling down program to 4°C. Primers were designed using the Roche Probe Design 2 software (Roche Diagnositcs) and purchased from TIB MOLBIOL (Berlin, Germany).

Insulin receptor isoform A was amplified using the following primers: forward TTT TCG TCC CCA GGC CAT, reverse CCACCGTCACATTCCCAAC. Insulin receptor isoform B was amplified using primers: forward TTT CGT CCC CAG AAA AAC CTC T, reverse CCA CCG TCA CAT TCC CAA C. Both reactions used 5′ 6-FAM phosphoramidite-TCG CCA AGG GAC CTG CGT T-BBQ (4,4-Bis-[2-butyloctyloxy]-p-quaterphenyl) as a probe. As an internal control, we used these PCRs to calculate the contribution of insulin receptor isoform A to the total insulin receptor content of three human tissues: In HepG2 hepatoma cells, 69% of the insulin receptor was isoform A, while in differentiated human adipocytes and in skeletal muscle, only 30% was isoform A. These values are comparable to values reported for these tissues in the literature [8]. p27^kip1^ (CDKN1B) was amplified with the primers: forward GAG AGC CAG GAT GTC AGC G, reverse TTG TTT TGA GTA GAA GAA TCG TCG GT. For this reaction, 5′ 6-FAM phosphoramidite-CCT TTA ATT GGG GCT CCG GCT AAC T-BBQ was used as a probe. The other reactions used standard Roche probes and the following primers: IGF-1 receptor forward TCA GCG CTG CTG ATG TGT, reverse GGC TCA TGG TGA TCT TCT CC; Insulin receptor substrate (IRS)-1 forward GCC TAT GCC AGC ATC AGT TT, reverse TTG CTG AGG TCA TTT AGG TCT TC; IRS-2 forward TGA CTT CTT GTC CCA CCA CTT, reverse CAT CCT GGT GAT AAA GCC AGA.

For immunohistochemistry, slides were deparaffinized, rehydrated and immersed in 3% hydrogen peroxide solution. Antigen retrieval was accomplished by slide boiling in citrate buffer.p27^kip1^ protein was detected using Vectastain Elite ABC kit (Vector Laboratories, Burlingame, CA, USA). After blocking endogenous avidin/biotin by the Vector blocking kit (Vector) and unspecific staining by incubation with normal serum (horse, ABC kit) a monoclonal mouse clone SX53G8 antibody (Dako, (Santa Barbara, CA, USA) was used in a dilution of 1∶150 at 4°C over night incubation. The secondary antibody was used according to the manufacturer's recommendations and DAB (Vector) was used for visualization. All slides were counterstained with Mayer's hematoxylin. Tonsil tissue served as positive control. Staining was quantified according to semiquantitative immunohistochemistry reference scale ranging from 0–300 as described in [19].

For statistical analyses, JMP 9.0 (SAS Institute, Cary, NC, USA) was used. Not normally distributed data were logarithmically transformed prior to statistical analysis. Differences between two groups were tested by Student's t-test. Differences between multiple groups were tested by ANOVA followed by Tukey Kramer post-hoc tests. Correlations were analyzed using linear regression analysis. Results with p<0.05 were considered statistically significant.

## Results

**Figure 1:**
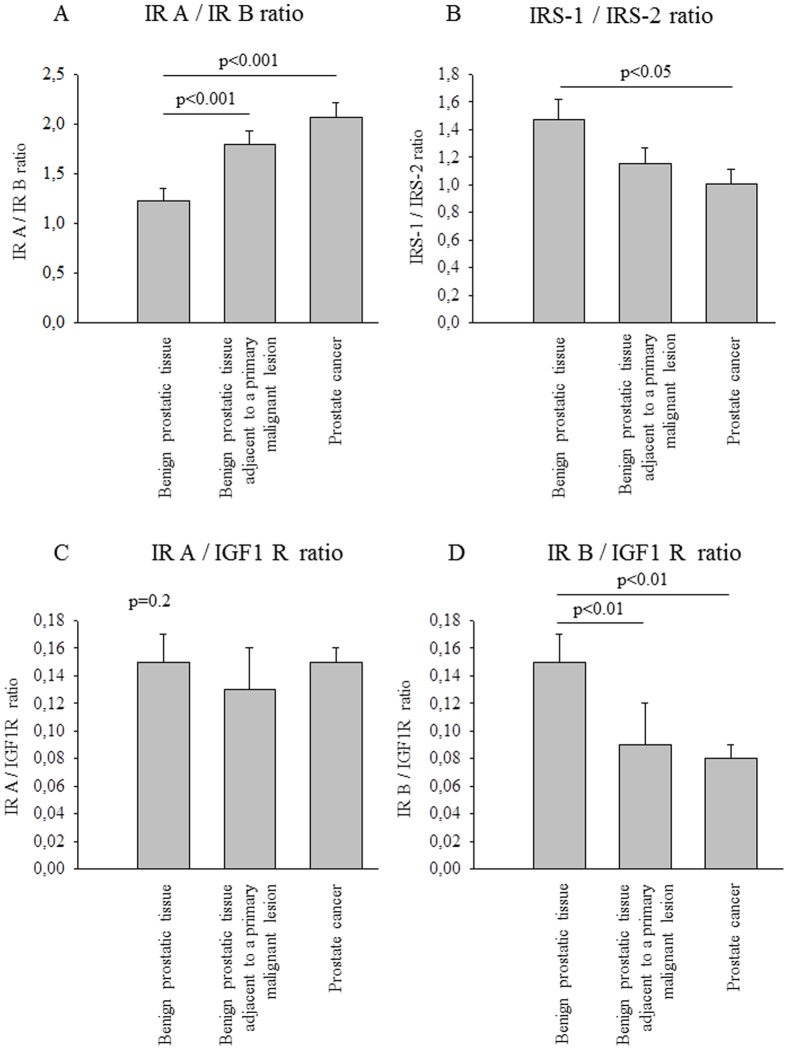
Proportion of the analyzed RNA expressions. Bars represent means + SEM., N = 88. Data were log*e*-transformed prior to statistical analysis. When comparison between groups by ANOVA resulted in statistically significant differences, Tukey Kramer post-hoc test was performed. Statistically significant results of this test are given in the figures. (A) Insulin receptor isoform A/Insulin receptor isoform B ratio. ANOVA p = 0.0002. (B) IRS-1/IRS-2 ratio. ANOVA p = 0.0426. (C) Insulin receptor isoform A/IGF 1 receptor ratio. ANOVA p = 0.2. (D) Insulin receptor isoform B/IGF 1 receptor ratio. ANOVA p = 0.0016. ANOVA – analysis of variance; IGF – insulin-like growth factor; IR – insulin receptor; IRS – insulin receptor substrate; RNA – ribonucleic acid; SEM – standard error of the mean.

To evaluate relative expression levels, we calculated ratios between the two IR isoforms, between each IR isoform and the IGF-1 receptor as well as between IRS 1 and 2. Comparing benign tissue with prostate cancer, IR isoform A to B ratio was significantly different in favor of isoform A in cancer compared to benign tissue (Figure 1 A). IRS-1 to IRS-2 ratio was altered with relatively higher IRS-2 expression (Figure 1 B). For both ratios, histological benign tissue adjacent to cancer was significantly different to benign tissue but not to prostate cancer (Figures 1 A and B). While there were no differences between groups in the IR isoform A to IGF-1 receptor ratio (Figure 1 C), IR isoform B to IGF-1 receptor ratio was significantly lower in cancer and adjacent tissue compared to benign prostate (Figure 1 D). When repeating the analyses in the subgroup of all patients without diabetes, all differences reported for the whole group remained significant (all post-hoc p<0.05, data not shown). Additionally, in this subgroup the IR isoform A to IGF-1 receptor ratio was significantly lower in histological benign tissue adjacent to cancer compared to the other two tissue types (ANOVA p = 0.0099, post-hoc p<0.05).

**Figure 2:**
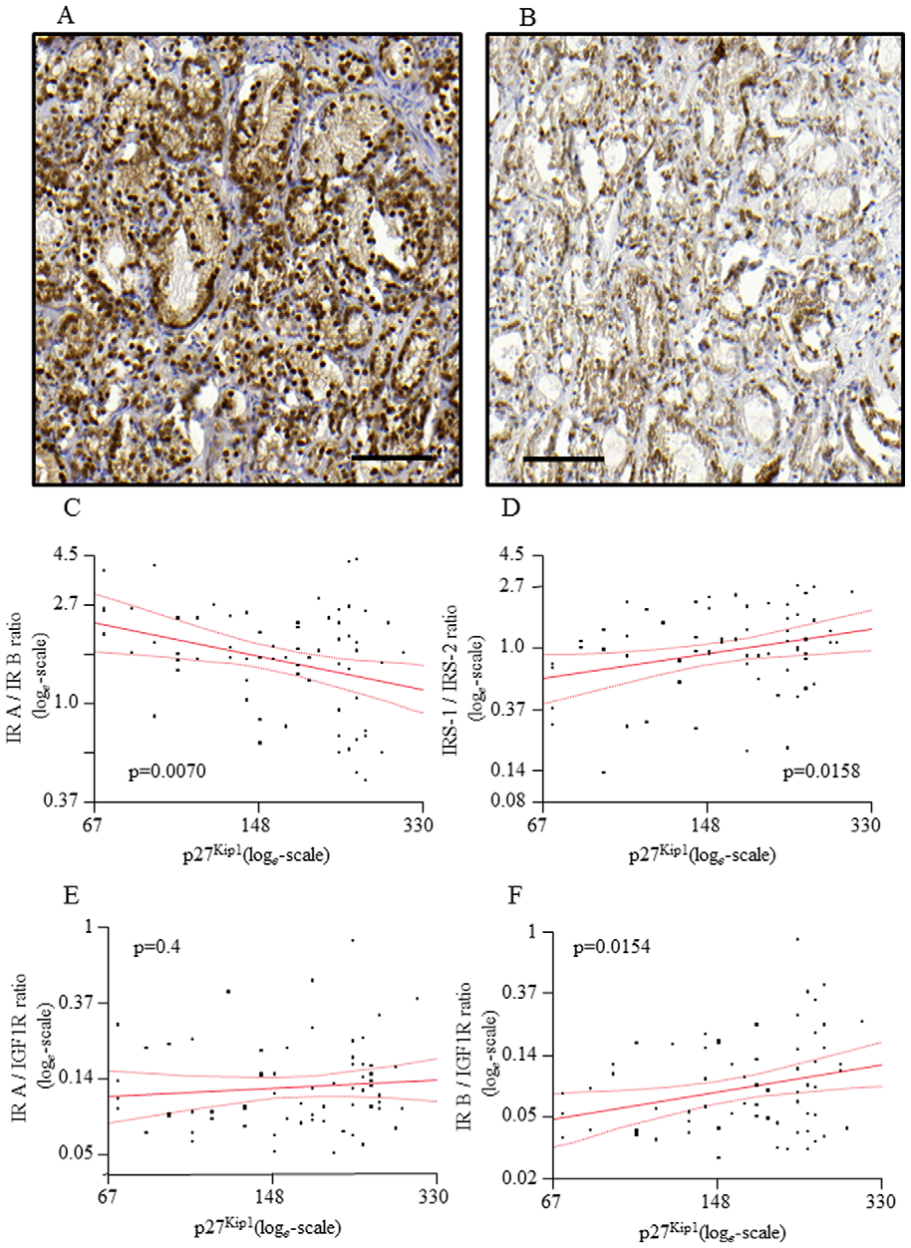
Ratios of investigated parameters and association to p27^Kip1^expression. (A) and (B): Representative staining for p27^Kip1^, A: a prostate cancer sample with strong staining. B: a prostate cancer sample with weak staining. Bar = 100 µm. (C)–(F): Gene expression ratios are blotted against quantification of p27^Kip1^ staining. Lines represents fit line ± Confidence Interval, N = 69. (C) Insulin receptor isoform A/Insulin receptor isoform B ratio. (D) IRS-1/IRS-2 ratio. (E) Insulin receptor isoform A/IGF 1 receptor ratio. (F) Insulin receptor isoform B/IGF 1 receptor ratio. Data were log*_e_*-transformed prior to statistical analysis. Correlations were analyzed by linear regression analysis. IRS – Insulin receptor substrate; IGF – Insulin-like growth factor.

In immunohistochemistry, expression of p27^Kip1^ was homogenously distributed along the glandular rings (Figure 2 A and B). In most samples a nuclear staining was present, while only in case of strong staining an additional cytoplasmic fraction was detected. Inflammatory cells and vessels stained positive but were not considered for quantitative evaluation. Samples were available for immunohistochemistry and staining was successful in 68 samples. There was a significant negative correlation of p27^Kip1^ with IR isoform A to B ratio (Figure 2 C). For the IRS-1 to IRS-2 ratio, we detected a significant positive correlation with pf27^Kip1^ (Figure 2 D). While p27^Kip1^ was positively correlated with IR isoform B to IGF-1 receptor ratio (Figure 2 F), no correlation with IR isoform A to IGF-1 receptor ratio was found (Figure 2 E).

In 55 samples, we also quantified expression of p27^Kip1^(CDKN1B) on the RNA level and detected a positive correlation to the immunohistochemical protein expression data (p = 0.0306). As with the protein data, p27^Kip1^ RNA was significantly negatively correlated to the IR isoform A to B ratio (p = 0.0069). For p27^Kip1^ RNA, there was a trend towards positive correlation to IRS-1 to IRS-2 ratio (p = 0.0675) as well as a trend towards positive correlation with the IR isoform A to IGF-1 receptor ratio (p = 0.0862). No correlation with IR isoform B to IGF-1 receptor ratio was found (p = 0.7).

In the prostate cancer samples, none of the analyzed mRNA expression ratios was associated with Gleason score, pre-operative PSA levels, or WHO's T- or N-stage (all p≥0.2).

When we compared samples of patients suffering from type 2 diabetes with such from patients without, we found no differences in IR isoform A to B ratio or in IRS 1/IRS 2 ratio (p = 1.0 and 0.7, respectively). IGF-1 receptor levels were, however, significantly lower in patients with type 2 diabetes (p = 0.0019).

## Discussion

We investigated the composition of molecules important for insulin signal transduction in prostate cancer in comparison to benign prostate tissue. Benign tissue was either derived from an entirely benign prostate or it was histologically benign tissue adjacent to prostate cancer. Interestingly, there were significant differences between malignant and benign prostate but not between tumor and adjacent tissue. In both, the ratio between IR isoforms A and B was different in favor of isoform A and the IRS-1 to IRS-2 ratio differed in favor of IRS-2. While the IR isoform A to B as well as the IRS-1 to IRS-2 ratios were not different in patients with type 2 diabetes, we found IGF-1 receptor to be reduced in this patient group.

As in many other cancers [8], we found higher expression of IR isoform A compared to isoform B. Accordingly, we found reduced levels of the cell cycle inhibitor p27^Kip1^ in samples with higher IR isoform A. Both insulin and IGF-II can activate this isoform; however there are differences in downstream signaling [20]. Furthermore, IGF-II binds to the IGF-1 Receptor and this receptor also prevailed the metabolically active IR isoform B in our prostate cancer samples. This was again associated with altered p27^Kip1^ levels. Thus, all our findings predict increased proliferative effects of insulin and IGF-II in prostate cancer. Of interest, specific antibodies for IR isoform A are under development for the treatment of cancer [8]. Based on our findings, these drugs might also offer therapeutic options in prostate cancer.

We found prostate cancer to overexpress IRS-2 over IRS-1. Interestingly, IRS-2 was identified as a positive regulator of metastasis in breast cancer, whereas IRS-1 may be a suppressor of metastasis [21], [22]. This role of IRS-2 in the pathogenesis of metastasis seems to be a general principal that applies for other malignancies as well [15], [21]. Since IRS-2 is downstream the IR for which the ligand, insulin, is markedly elevated in T2DM, our findings of higher IRS-2 in prostate cancer suggests increased risk for metastasis in men with diabetes. In line with this hypothesis, survival after diagnosis of prostate cancer is significantly shorter in patients with T2DM compared to such without [7].

The significantly lower IGF-1 receptor expression in samples from patients with type 2 diabetes is much unexpected to us. We are not aware of any reports on such a phenomenon in prostate or any other tissue. However, based on the relatively small number of patients with diabetes in our study, we could not analyze this finding separately in prostate cancer versus healthy prostate. Of course, this should be investigated in future studies, since reduced IGF-1 receptor availability in prostate cancer in patients with type 2 diabetes could make them more likely to respond to certain radio- or chemotherapies [23], [24], but might make them resistant to treatments that directly target the IGF-1 receptor [24].

One further surprising finding of our study is the characteristics of histologically benign tissue adjacent to prostate cancer. Even if this tissue appears as normal prostate tissue histologically, its composition of insulin signaling molecules is more similar to cancer than to healthy tissue. Comparable observations have been made in earlier studies as well [9], [10]. At least two different explanations are conceivable: The insulin signaling cascade in the whole prostate could be altered long before cancer develops with a second hit being necessary to trigger cancer emerging. Another possible explanation is that the cancer modifies its surrounding tissue and induces a tissue gradient of IR/IRS subtype expression. Despite being histologically benign, the adjacent tissue could therefore already be altered by the tumor and may eventually harbor tumorigenic potential. This phenomenon is present in other malignancies, for instance epigenetic changes are present as far as 4 cm away from breast cancer [25]. If the second hypothesis proves true in further studies, it could have great implications for the diagnosis of prostate cancer: even if the cancer focus would be missed in a prostate biopsy, altered IR/IRS composition in the specimen would indicate cancer within the organ and prompt further actions.

One limitation of our study is that we did not measure fasting insulin and fasting glucose levels in the patients from whom the investigated samples are derived. Major determinants of these parameters, namely age and body mass index (BMI) as well as prevalence of type 2 diabetes and use of diabetes drugs, did not differ between patient groups in our study. However, insulin and glucose were demonstrated to regulate IR isoform composition [8], [26], [27], and further studies should therefore address these parameters.

Furthermore, it would be very interesting to compare IR/IRS composition on the protein level in addition to the mRNA level. The available amount of tissue in our study was not enough for sufficient western blots. Moreover, we had access to only one sample out of each prostate. Studying multiple samples from a single organ with different proximity to the site of cancer could allow detailed studies on possible gradients of IR and IRS isoform composition from cancer to surrounding tissue. Such analyses could unravel the cause for our observation of similarities between prostate cancer and adjacent tissue.

Taken together, we found significant differences in the IR signaling cascade between benign prostate tissue and cancer. Histological benign tissue adjacent to prostate cancer showed expression patterns similar to the malignancies. Our findings suggest a role of the insulin signaling pathway in prostate cancer and can hence be relevant for both novel diagnostic as well as therapeutic approaches in this malignancy.
